# Structural and Functional Disruptions in Subcortical Vascular Mild Cognitive Impairment With and Without Depressive Symptoms

**DOI:** 10.3389/fnagi.2019.00241

**Published:** 2019-09-13

**Authors:** Hanqing Lyu, Jianjun Wang, Jinping Xu, Haotao Zheng, Xiaoyan Yang, Songjun Lin, Jianxiang Chen, Liuchang Zhou, Yuanming Hu, Zhouke Guo

**Affiliations:** ^1^Department of Radiology, Shenzhen Traditional Chinese Medicine Hospital/The Fourth Clinical Medical College, Guangzhou University of Chinese Medicine, Shenzhen, China; ^2^Department of Neurology and Psychology, Shenzhen Traditional Chinese Medicine Hospital/The Fourth Clinical Medical College, Guangzhou University of Chinese Medicine, Shenzhen, China; ^3^Institute of Biomedical and Health Engineering, Shenzhen Institutes of Advanced Technology, Chinese Academy of Sciences, Shenzhen, China

**Keywords:** vascular mild cognitive impairment, subcortical impairments, cerebral small vessel disease, subthreshold depression, resting-state functional magnetic resonance imaging, voxel-based morphometry

## Abstract

Many previous studies have revealed structural and functional abnormalities in patients with the subcortical vascular mild cognitive impairment (svMCI). Although depression symptoms were suggested to serve as a potential marker of conversion to dementia in patients with svMCI, whether these disruptions or other new findings will be identified in the svMCI comorbid with depression symptoms has not been established. In the current study, we combined voxel-based morphometry (VBM) and the resting-state functional magnetic resonance imaging (fMRI) to investigate the structural and functional disruptions in the svMCI with and without depression symptoms using a cohort of 18 svMCI with depression symptoms (svMCI+D), 17 svMCI without depression symptoms (svMCI−D), and 23 normal controls (NC). As a result, we identified significantly decreased gray matter density in the left parahippocampus (ParaHIPP.L), the right hippocampus (HIPP.R), and the right middle cingulate cortex (MCC.R) in both svMCI+D and svMCI−D compared to NC. Most importantly, we also identified increased gray matter density in the MCC.R accompanied by increased resting-state functional connectivity (RSFC) with right parahippocampus (ParaHIPP.R) in the svMCI+D compared to svMCI−D. Moreover, the gray matter density of MCC.R and ParaHIPP.L was correlated with cognitive impairments and depression symptoms in the svMCI, respectively. In conclusion, these results extended previous studies and added weight to considerations of depression symptoms in the svMCI. Moreover, we suggested that a processing loop associated with HIPP, ParaHIPP, and MCC might underlie the mechanism of depression symptoms in the svMCI.

## Introduction

The subcortical vascular mild cognitive impairment (svMCI) is a nonamnestic subtype of MCI, which is characterized by extensive white matter hyperintensities (WMH) and multiple lacunar infarctions on magnetic resonance imaging (MRI; Frisoni et al., 2002). Recently, more and more attention was paid to svMCI since it is regarded as a prodromal stage of subcortical vascular dementia (SVaD; Frisoni et al., 2002; Seo et al., 2010; Kim et al., 2011) but is potentially reversible by managing the risk factors and drug treatments (Ravaglia et al., 2006; Jak et al., 2009). Compared to other risk factors, depression symptoms were underestimated even though it is common in people with MCI (Palmer et al., 2007; Solfrizzi et al., 2007; Enache et al., 2011; Orgeta et al., 2015; Liu et al., 2019). Recently, a study reported that the prevalence of depression was highest in MCI (up to 18.8%) and was associated with different cognitive domains (Vloeberghs et al., 2018). In fact, depression symptoms, even at minimal severity, have been reported to be associated with progression from MCI to dementia (Gabryelewicz et al., 2007; Teng et al., 2007; Palmer et al., 2010; Rosenberg et al., 2013). Moreover, Kim et al. (2013) suggested that depression symptoms might serve as a potential marker of conversion to dementia in patients with svMCI. Therefore, it is important and useful to investigate the structural and functional disruptions in the svMCI with and without depression symptoms (svMCI+D and svMCI−D, respectively).

To date, too many previous studies have revealed structural and functional abnormalities in patients with svMCI regardless of depression symptoms. Specially, gray matter volume reductions in a wide range of cortical regions (e.g., superior and middle frontal gyrus, medial prefrontal gyrus, superior and inferior temporal gyrus), as well as in the subcortical regions (e.g., hippocampus, parahippocampal gyrus, thalamus, and caudate; Yi et al., 2012; Li et al., 2017) were reported in the svMCI. Besides gray matter reductions, cortical thinning in the inferior frontal and orbitofrontal gyri, the anterior cingulate, the insula, the superior temporal gyrus, and the lingual gyrus were also identified in the svMCI (Seo et al., 2010; Lee et al., 2018). Additionally, svMCI patients also presented decreased functional amplitude of spontaneous low-frequency oscillations in the default mode network (Yi et al., 2012), altered functional connectivity of the posterior cingulate cortex (Ding et al., 2015), and altered functional connectivity of the thalamus (Zhou et al., 2016). However, whether these disruptions or other new findings will be identified in the svMCI comorbid with depression symptoms has not been established.

In the current study, we combined voxel-based morphometry (VBM) and the resting-state functional magnetic resonance imaging (fMRI) to investigate the structural and functional disruptions in the svMCI with and without depression symptoms using a cohort of 18 svMCI+D, 17 svMCI−D, and 23 normal controls (NC).

## Materials and Methods

### Participants

We recruited 40 right-handed patients with svMCI from the Department of Encephalopathy and Psychology in Shenzhen Traditional Chinese Medicine Hospital. The diagnosis criteria of svMCI was performed by two experienced neurologists in consensus according to the modified Petersen’s criteria (Petersen, 2004; Yi et al., 2015; Jia et al., 2016), which included the following: (1) patients who reported cognitive impairments involving memory and/or other cognitive domains lasting for at least 3 months; (2) objective cognitive impairments, although not meeting the Diagnostic and Statistical Manual of Mental Disorders, Fourth Edition (DSM-IV) criteria for dementia; (3) a clinical dementia rating (CDR) = 0.5; (4) with a Montreal Cognitive Assessment (MOCA) score <26; (5) normal or minimum impairments of daily life activities (Yi et al., 2012); and (6) subcortical vascular causes of the cognitive impairments according to: (a) at least three supratentorial subcortical small infarcts (diameters ranging from 3 to 20 mm) with or without white matter lesions (WMLs) of any degree, or moderate to severe WML (score ≥2 according to the Fazekas rating scale; Fazekas et al., 1987) with/without small infarct, or one or more strategically located subcortical small infarcts in the caudate nucleus, globus pallidus, or thalamus in the T2 fluid-attenuated inversion recovery (FLAIR) images; (b) absence of cortical and watershed infarcts, hemorrhages, hydrocephalus, and WMLs with specific causes (e.g., multiple sclerosis); and (c) no hippocampal or entorhinal cortex atrophy (scored 0 according to the medial temporal lobe atrophy scale of Scheltens; Scheltens and van de Pol, 2012). All the patients were native Chinese, with ages from 50 to 70 years. Moreover, all svMCI patients underwent a global mental functioning through the MOCA. The severity of depression symptoms was assessed using the 17-item Hamilton Depression Rating Scale (HAMD). Patients with HAMD-17 scores 7–17 were classified into the svMCI+D, and those who scored less than 7 were classified into the svMCI−D (Riedel et al., 2010; Zimmerman et al., 2013; Pavlovic et al., 2016).

We also excluded patients who had: (1) severe aphasia and physical disabilities that may preclude completion of neuropsychological testing; (2) disorders with use of medications that may affect cognition; (3) a score of HAMD >17; (4) schizophrenia; (5) strokes within 3 months; (6) inherited or inflammatory small vessel disease; (7) clinically significant diseases, such as gastrointestinal, renal, hepatic, respiratory, infectious, endocrine, or cardiovascular system disease; (8) cancer, alcoholism, and drug addiction; (9) known hypersensitivity to celery; and (10) inability to undergo a brain MRI. To ensure consistent application of the criteria and minimize diagnostic variability, we used a central neuroimaging reader to determine eligibility.

Twenty-three age-, gender-, and education-matched NC were also included in the study, who had no history of any neurological or psychiatric disorders, no cognitive complaints, and no abnormalities in their conventional brain MRI images. During the research, three svMCI patients were excluded for personal reasons, and two controls and two svMCI patients were excluded for visible head motion. Thus, 18 svMCI+D patients, 17 svMCI−D patients, and 23 NC were included in the final analysis. The demographic and clinical data are shown in Table 1. All subjects gave written informed consent, and the study was approved by the Institutional Review Board of the Shenzhen Traditional Chinese Medicine Hospital.

**Table 1:** Demographic data and clinical measures.

Groups	svMCI+D	svMCI−D	NC	*F*-value	*p*-value
Subjects	18	17	23	-	-
Age	61.66 ± 6.49	65.82 ± 7.16	61.91 ± 4.86	2.601	0.083
Gender	8:10	9:8	9:14	-	0.685
Education	7.66 ± 3.77	8.70 ± 3.83	9.82 ± 3.55	1.728	0.187
MOCA	18.83 ± 1.68	20.23 ± 2.33	27.91 ± 1.04	171.88	<0.001^a,b,c^
HAMD	11.38 ± 2.50	4.88 ± 1.31	2.30 ± 1.55	126.124	<0.001^a,b,c^

*ANOVA showed significant differences in the Montreal Cognitive Assessment (MOCA) and Hamilton Depression Rating Scale (HAMD) scores. a–c: *post hoc* analysis revealed the source of ANOVA (a: svMCI+D vs. NC, b: svMCI−D vs. NC, and c: svMCI+D vs. svMCI−D). Abbreviation: ANOVA, analysis of variance*.

### MRI Acquisition

MRI scanning was conducted on a 3T scanner (GE medical system, MR750). The scanning parameters of the fMRI images were as follows: repetition time/echo time ratio = 2,000/35 ms, flip angle = 90°, matrix size = 64 × 64, slice thickness = 4 mm, voxel size = 4 × 4 × 4 mm^3^, and volumes = 240. The scanning parameters of the T1 images were as follows: repetition time/echo time ratio = 8.656/3.22 ms, inversion time = 450 ms, flip angle = 12°, matrix size = 256 × 256, slice thickness = 1 mm, voxel size = 1 × 1 × 1 mm^3^, and sections = 152. The scanning parameters of the T2 FLAIR images were as follows: repetition time/echo time ratio = 9,000/92.544 ms, flip angle = 160°, matrix size = 256 × 224, slice thickness = 6.5 mm, and voxel size = 0.47 × 0.47 × 6.5 mm^3^.

### VBM Analysis

VBM analysis was performed to determine brain structural differences among the three groups. The structural MRI images were preprocessed using the DPABI toolbox^1^. The steps were as follows: (1) each T1 image was segmented into gray matter, white matter, and cerebrospinal fluid using a fully automated algorithm within DPABI; (2) all images were subsequently transformed to the Montreal Neurological Institute (MNI) space using DARTEL normalization; (3) the resulting images were smoothed with a Gaussian kernel of 6-mm full width at half maximum (FWHM = 6 mm); and (4) the modulated images were used to calculate the gray matter density and were corrected for total intracranial volume.

### Resting-State fMRI Data Preprocessing

Preprocessing of the resting-state fMRI data was also performed using DPABI. For each participant, the preprocessing steps were as follows: (1) the first 10 volumes of each functional time series were discarded to allow for magnetization equilibrium; (2) the slice times for the remaining 230 images were corrected, and they were realigned to the first volume to account for head motion (subjects with head motion exceeding 3 mm in any dimension or 3° of angular motion through the resting-state run were removed); (3) all data were spatially normalized to the MNI template and resampled to 3 × 3 × 3 mm^3^; (4) spatial smoothing was performed using FWHM = 6 mm; (5) linear and quadratic trends were removed; (6) the Friston 24-parameter model (Friston et al., 1996) was utilized to regress out head motion effects from the realigned data (Satterthwaite et al., 2013; Yan et al., 2013); (7) the white matter, cerebrospinal fluid, and global signals were regressed out; (8) temporal band-pass filtering (0.01–0.1 Hz) was performed; and (9) “scrubbed” was used to eliminate the bad images, captured before two time points and after one time point, which exceeded the preset criteria [frame displacement (FD), <0.5] for excessive motion (Power et al., 2012).

### Statistical Analysis of Demographic and Clinical Measures

Demographic and clinical measures were analyzed using one-way analysis of variance (ANOVA) with Bonferroni correction, while gender was analyzed using *χ*^2^ using SPSS 19 (SPSS Inc., Chicago, IL, USA). The source of the differences between the means of the three groups was examined by *post hoc* *t*-tests.

### Group Difference of Gray Matter Density

Group difference of gray matter density was tested using analysis of covariance (ANCOVA) among svMCI+D, svMCI−D, and NC, while controlling for age, education, and gender. Gaussian random field (GRF) corrections were used for all multicomparison correction (a voxel-level of *p* < 0.001 and a cluster-level of *p* < 0.05). Then, the mean gray matter density in the altered clusters was calculated, separately, in the three groups. Group differences were compared among the three groups using ANCOVA and *post hoc* *t*-tests between any two groups using SPSS.

### Resting-State Functional Connectivity (RSFC) Analyses

To investigate the corresponding functional connectivity patterns of each altered brain region, we also performed RSFC analysis among the three groups. The RSFC of each altered region was defined by Pearson correlation coefficients between the mean time series of each seed region and that of each voxel in the rest of the brain. We used the binary gray matter mask in SPM before computing the whole brain RSFC. Correlation coefficients were converted to *z*-values using Fisher’s *z* transformation to improve normality.

Group difference of the RSFC was tested using ANCOVA among svMCI+D, svMCI−D, and NC, while controlling for age, education, and gender. GRF corrections were used for all multicomparison correction (a voxel level of *p* < 0.001 and a cluster-level of *p* < 0.05).

Moreover, we calculated the mean RSFC of the regions that showed significantly altered RSFC with seed regions in the three group. Group differences were compared among the three groups using ANCOVA and *post hoc* *t*-tests between any two groups using SPSS. To exclude the effects of global signal, we reanalyzed the mean RSFC using resting-state fMRI data without global signal regression.

### Correlation Analyses

We also performed correlation analysis between neuroimaging measures (mean gray matter density and RSFC) and the clinical measures (MOCA and HAMD scores) to further explore whether neuroimaging indices were related to the symptom level, respectively, in svMCI+D and svMCI−D while controlling for age, gender, and education. The significance level was set at *p* < 0.05.

## Results

### Demographic Data and Clinical Measures

The demographic and clinical measures were summarized in Table 1. No significant differences were found among the three groups in age, gender, and education (*p* > 0.05). The MOCA scores of the svMCI+D were the smallest in the three groups, representing the heaviest cognitive impairment, while the HAMD scores of the svMCI+D were the highest in the three group, representing the heaviest depression.

### VBM Results

**Figure 1:**
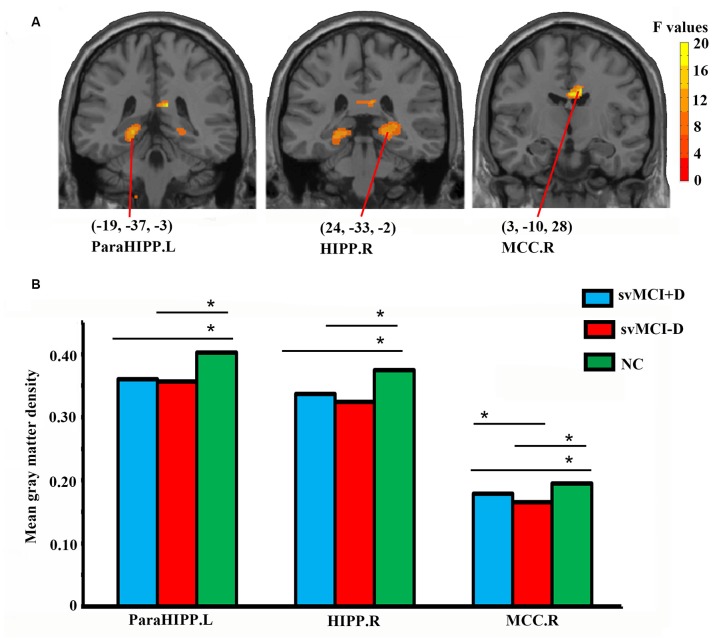
Abnormal gray matter density in the whole brain **(A)** and regional-level **(B)** analyses among subcortical vascular mild cognitive impairment (svMCI) with depression symptoms (svMCI+D), svMCI without depression symptoms (svMCI−D), and normal control (NC). Analysis of covariance (ANCOVA), *post hoc* *t*-tests and gaussian random field (GRF) correction (voxel level *p* < 0.001, cluster level *p* < 0.05) were used. *Significant at *p* < 0.05. Abbreviations are listed in Table 2.

Significantly altered gray matter density was identified in the left parahippocampus (ParaHIPP.L), the right hippocampus (HIPP.R), and the right middle cingulate cortex (MCC.R) among the three groups (Figure 1A and Table 2).

**Table 2:** Abnormal gray matter density among svMCI with depression symptoms (svMCI+D), svMCI without depression symptoms (svMCI−D), and normal control (NC) using ANCOVA.

Comparison	Brain regions	Cluster size	Peak intensity	Peak MNI coordinates
svMCI+D and svMCI−D and NC	ParaHIPP.L	652	14.0391	(−19, −37, −3)
	HIPP.R	614	11.5422	(24, −33, −2)
	MCC.R	944	19.88	(3, −10, 28)

*Abbreviations: L, left; R, right; ParaHIPP, parahippocampus; HIPP, hippocampus*.

For the three brain regions that showed significantly altered gray matter density among the three groups, group differences of regional mean gray matter density were investigated using *post hoc* *t*-tests. Both the svMCI+D and svMCI−D group showed decreased mean gray matter density in the ParaHIPP.L, HIPP.R, and MCC.R compared to the NC. Moreover, we also found significantly increased mean gray matter density of the MCC.R in the svMCI+D compared to the svMCI−D (Figure 1B).

### RSFC Results

**Figure 2:**
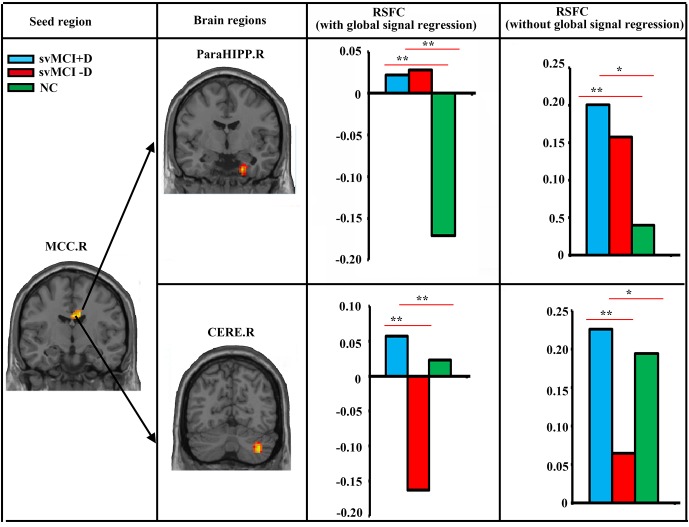
Abnormal resting-state functional connectivity (RSFC) among svMCI+D, svMCI−D, and NC. ANCOVA, *post hoc* *t*-tests and GRF correction (voxel level *p* < 0.001, cluster level *p* < 0.05) were used. The mean RSFC of the altered brain regions were calculated both with and without global signal regression in the three groups, and *post hoc* *t*-tests were performed between the RSFC of any two groups. **Significant at *p* < 0.001. *Significant at *p* < 0.05.

We found significantly increased RSFC between the MCC.R and right parahippocampus (ParaHIPP.R) in both the svMCI+D and svMCI−D groups compared to the NC (Figure 2). However, compared to NC, the RSFC between the MCC.R and right cerebellum (CERE.R) was increased in the svMCI+D but decreased in the svMCI−D. The results were similar with and without global signal regression.

### Correlation Results

**Figure 3:**
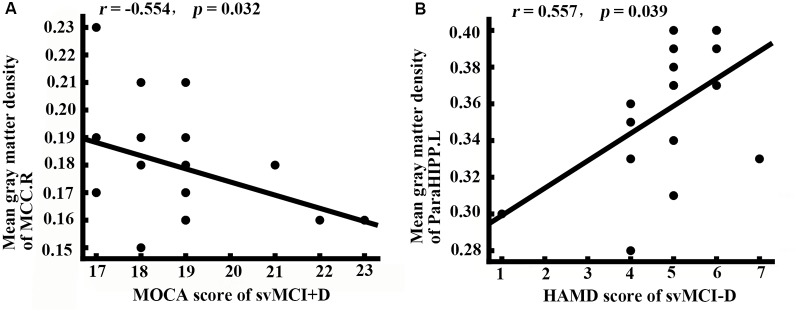
Correlation analysis. **(A)** Correlation analysis showed that the mean gray matter density of right middle cingulate cortex (MCC.R) was correlated with the montreal cognitive assessment (MOCA) score in svMCI+D patients (*p* < 0.05). **(B)** Correlation analysis showed that the mean gray matter density of ParaHIPP.R was correlated with the HAMD score in svMCI−D patients (*p* < 0.05).

While controlling for age, gender, and education, the MOCA scores of the svMCI+D were significantly correlated with the mean gray matter density of the MCC.R, and the HAMD scores of the svMCI−D were positively correlated with the mean gray matter density of the ParaHIPP.L (Figure 3).

## Discussion

Combining the VBM and RSFC, we identified significantly decreased gray matter density in the ParaHIPP.L, HIPP.R, and MCC.R in both the svMCI+D and svMCI−D compared to NC. Most importantly, we also identified increased gray matter density in the MCC.R accompanied by increased RSFC with ParaHIPP.R in the svMCI+D compared to the svMCI−D. Moreover, the gray matter density of MCC.R and ParaHIPP.L were correlated with cognitive impairments and depression symptoms in the svMCI, respectively.

We identified significantly decreased gray matter density in the ParaHIPP.L and the HIPP.R in both the svMCI+D and svMCI−D compared to NC. Similar with our results, the structural disruptions of the HIPP.R, such as gray matter volume atrophy (Fein et al., 2000; Li et al., 2017), hippocampal subfield volume atrophy (Li et al., 2016), hippocampal shape deformities (Kim et al., 2014, 2015), and altered structural covariance of the hippocampal subfields (Wang et al., 2018), were identified in several neuroimaging studies in the svMCI. Recently, a VBM study also reported gray matter volume reductions in both the HIPP.R and the ParaHIPP.L in the svMCI compared to NC (Li et al., 2017). In addition, the svMCI patients also showed elevated susceptibility values within the HIPP.R, which was negatively correlated with memory *z*-scores (Sun et al., 2017). Since the HIPP and ParaHIPP are reported to be involved in memory consolidation (Runyan et al., 2019), the decreased gray matter density in these two regions might be associated with memory loss in the svMCI. However, there were no memory measurements in our study, which weakens our interpretation.

Most importantly, the gray matter density in the MCC.R was decreased in both the svMCI+D and svMCI−D compared to NC, but it was increased in the svMCI+D compared to the svMCI−D. On the one hand, the disruptions of MCC.R were in line with previous results, which showed significantly disrupted intramodular connectivity in the middle cingulate cortex (MCC) in the svMCI (Yi et al., 2015) and reduced nodal efficiency in the MCC in late-life depression with comorbid amnestic MCI (Li et al., 2015). On the other hand, the finding of increased gray matter density in the MCC in the svMCI+D compared to the svMCI−D is innovative and noteworthy. Moreover, further correlation analysis showed that the gray matter density of MCC.R is negatively correlated with MOCA scores of the svMCI+D, indicating that the higher gray matter density of the MCC.R, the more severe the cognitive impairment in the svMCI+D. Taken together, our results offered extra evidence to support the hypothesis that depression itself can primarily cause and/or worsen cognitive deficits in the MCI (Palmer et al., 2010). More specifically, we suggested that the following processing loop might underlie the mechanism of depression symptoms in the svMCI: the svMCI showed decreased gray matter density in the HIPP.R and ParaHIPP.L, which might initially cause depression symptoms (association between the gray matter density of the ParaHIPP.L and HAMD scores in the patients with svMCI−D) and induce increased RSFC between the ParaHIPP.R and MCC.R, resulting in increased gray matter density of the MCC.R as time goes by, which finally worsens the cognitive impairments (association between the gray matter density of the MCC.R and MOCA scores in the patients with svMCI+D) and decreases the gray matter density of the HIPP.R and ParaHIPP.L in return. However, a longitudinal analysis that can provide more direct evidence is needed to confirm our suggestion.

Moreover, two major limitations should be stressed in the current study. First, all patients had subcortical small infarcts (<2 mm) and/or WML around the ventricle or randomly distributed in the whole brain. Since these lesions were relatively small and distributed randomly, it was extremely difficult to assess the location and volume of all the lesions and remove all of them. We processed the data as usual and did not control or consider the effects of these lesions. Thus, we cannot exclude the potential effects of lesions on our results. Second, the sample size is relatively small. A larger sample size is further needed to validate our results.

## Conclusion

In conclusion, our results extended previous studies and added weight to considerations of depression symptoms in the svMCI. Moreover, we suggested that a processing loop associated with HIPP, ParaHIPP, and MCC might underlie the mechanism of depression symptoms in the svMCI.

## Data Availability

The datasets generated for this study are available on request to the corresponding author.

## Ethics Statement

The studies involving human participants were reviewed and approved by the Institutional Review Board of the Shenzhen Traditional Chinese Medicine Hospital. The patients/participants provided their written informed consent to participate in this study.

## Author Contributions

YH and ZG designed the research. HL, JW, HZ, XY, SL, JC and LZ participated in the data collection. HL, JX and HZ analyzed the data. HL and JW wrote the manuscript. All authors read and approved the final manuscript.

## Conflict of Interest Statement

The authors declare that the research was conducted in the absence of any commercial or financial relationships that could be construed as a potential conflict of interest.

## Footnotes

^1^http://rfmri.org/dpabi
